# Zur seelischen Gesundheit von Studierenden – Eine Bestandsaufnahme aus der Westfälischen Hochschule

**DOI:** 10.1007/s11613-023-00827-1

**Published:** 2023-06-12

**Authors:** Eva-Maria Lewkowicz

**Affiliations:** Westfälische Hochschule, August-Schmidt-Ring 10, 45665 Recklinghausen, Deutschland

**Keywords:** Seelische Gesundheit, Covid-19, Hochschulen, Mental health, Covid-19, Higher education institutions

## Abstract

Dieser Praxisbericht geht der Frage nach, wie tiefgreifend und nachhaltig die Corona-Krise die (seelische) Gesundheit von Studierenden der Westfälischen Hochschule verändert hat, mit welchen Strategien sie darauf reagiert haben und wie sie sich heute zurecht finden. Basis ist eine Befragung unter den Studierenden im Wintersemester 2022/23. Im Ergebnis zeigt sich, dass die Folgen der Corona-Krise, vor allem aber auch die aktuelle geopolitische und wirtschaftliche Situation zu einer großen Verunsicherung geführt haben. Aus den Umfrageergebnissen werden Ansatzpunkte für die Beratung und Unterstützung der Studierenden abgeleitet.

## Abstract (en)

This practical report explores the question of how profoundly and sustainably the Corona crisis changed the (mental) health of students at the Westphalian University of Applied Sciences, what strategies they used to react to it and how they find their way today. The basis is a survey of students in the winter semester 2022/23. The results show that the consequences of the Corona crisis, but above all the current geopolitical and economic situation, have led to great uncertainty. Starting points for advising and supporting students are derived from the survey results.

## Einleitung

Als die Hochschulen im April 2020 Corona-bedingt in die Distanz-Lehre wechselten, war allem Schrecken der Epidemie zum Trotz auch Aufbruchsstimmung zu spüren. Neue Lehrformen wurden in Eilgeschwindigkeit entwickelt, Videokonferenzsysteme in die Lehrplattformen der Hochschulen integriert, Flipped Classroom Konzepte entworfen. Vor allem haben sich auch die Studierenden bereitwillig und neugierig auf das Neue eingelassen und damit gearbeitet. Doch schon in der ersten Prüfungsphase nach dem Sommersemester 2020 war das Hochgefühl einer deutlichen Ernüchterung gewichen: Das Engagement und die Leistungen der Studierenden hatten im Mittel stark nachgelassen, die Prüfungs-Ergebnisse deuteten darauf hin, dass einige von den neuen Lehrformen inspiriert ihre Leistungen steigern konnten, das Gros sich mehr schlecht als recht durchkämpfte, viele auch vollständig abgehängt zu werden drohten. In persönlichen Gesprächen und einem wöchentlichen online-Jour fixe im Sommersemester 2020 zur Unterstützung von 33 Masterstudierenden war mehrfach von dramatischen Corona-Krankheitsverläufen und Todesfällen in der Familie berichtet worden, Studierende mit Kindern standen unter hohem Druck, die Selbstorganisation der Studierenden kam an ihre Grenzen.

Die ersten regulären Präsenzveranstaltungen fanden nach dem Höhepunkt der Corona-Krise im Wintersemester 2021/22 noch unter Corona-Restriktionen statt: Impf- oder Teststatus-Kontrollen am Eingang in die Gebäude, Abstands- und Maskenpflichten im Hörsaal, keinerlei Campus-Leben aufgrund der geschlossenen Mensa, Aufenthaltsverbote in den Gängen und Zugangsbeschränkungen zur Bibliothek. Die Zahl der Studierenden, die an den Veranstaltungen teilnahmen, hatte sich stark reduziert, die Prüfungsleistungen weiter verschlechtert.

Seit dem Sommersemester 2022 läuft die Lehre wieder vollständig in Präsenz. Als im Wintersemester 2022/23 die ersten Erstsemester ihren Studienstart in Präsenz erleben konnten, die großen Anfänger-Vorlesungen voll waren, wieder „Ersti-Partys“ stattfinden konnten, war die Erleichterung groß. Und doch ist nicht zu übersehen: Viele Studierende kommen nicht mehr zu den Veranstaltungen, melden sich nicht zu Prüfungen an. Im Fachbereich Wirtschaftsrecht betrifft dies 20–30 % der Studierenden, insbesondere der höheren Semester. In den Veranstaltungen ist es im Erleben der Autorin schwieriger geworden, einen wissenschaftlichen Diskurs anzuregen, da sich die Studierenden en gros zurückhaltender verhalten und auf weniger sicheres Wissen zurückgreifen können. In den Sprechstunden kommen mehr persönliche Probleme zur Sprache als in den Jahren zuvor, häufiger waren Hinweise auf depressive Verstimmungen herauszuhören, häufiger wurde von Panikattacken und Prüfungsangst berichtet.

Um diese persönlichen Eindrücke abzugleichen und ein breiteres und gleichzeitig tieferes Bild des seelischen Erlebens der Studierenden zu gewinnen, wurde am 7. Dezember 2022 ein Fragebogen an die Studierenden aller Fachbereiche der Westfälischen Hochschule versandt. Dieser beinhaltete 28 Fragen, von denen bei 9 Fragen ein offenes Textfeld die Möglichkeit eingeräumt hat, persönliche Kommentare abzugeben. Vom 7. Dezember 2022 bis zum 28. Februar 2023 haben 753 Studierende die Seite mit dem Fragebogen aufgerufen, 674 an der Befragung teilgenommen und 596 Studierende den Fragebogen vollständig ausgefüllt. Bei ca. 8034 Studierenden der Westfälischen Hochschule (Stand WS 2021/22) entspricht dies einem Rücklauf von 8,4 % für die Teilnehmer und 7,4 % für die Teilnehmer, die den Fragebogen vollständig ausgefüllt haben.

Da dieser Fragebogen an alle Fachbereiche^1^ verschickt wurde und die Hochschule einen starken MINT-Fokus hat, ist das Antwortverhaltung nicht auf Hochschulen mit einer anderen Fächerstruktur generalisierbar. Auch die Lage der Hochschule im nördlichen Ruhrgebiet mit seinen sozialen Besonderheiten wird das Antwortverhalten beeinflusst haben. Zudem ist davon auszugehen, dass relativ mehr Studierende der WHS, die die Autorin als Professorin persönlich kennen, den Fragebogen ausgefüllt haben, dass also im Fachbereich Wirtschaftsrecht in Recklinghausen die Beteiligung höher war als im Durchschnitt der Fachbereiche insgesamt. Entsprechend diesen Verzerrungen ist der Anspruch dieses Praxisberichts nicht, den Status quo im Sinne verallgemeinerbarer Aussagen zu erfassen oder eine statistisch valide Auswertung vorzulegen. Vielmehr soll ein Stimmungsbild gezeichnet werden, das auf freiwilliger und anonymer Selbstauskunft beruht und Ansatzpunkte für die Unterstützung der Studierenden auf persönlicher und organisatorischer Ebene bietet.

## Ausgangssituation

Die gesundheitlichen Auswirkungen der Corona-Pandemie waren und sind in der Gesellschaft ungleich verteilt. Insbesondere ergaben sich ein höheres Risiko, sich anzustecken, einen schweren Verlauf zu erleben und eine höhere Sterblichkeit, für Menschen mit niedrigem sozialem Status und einem geringen Bildungsniveau (Hoebel et al. 2022; Knöchelmann und Richter 2021). Diese Befundlage ist „zu einem guten Teil als direkte Folge der sozialen Benachteiligung anzusehen“ (Heisig 2021, S. 334). Für Studierende kann allgemein von einer eher angespannten finanziellen Situation ausgegangen werden. Im Jahr 2016 lag das Mittel der Einnahmen von Studierenden in Deutschland bei knapp 920 €/Monat (Middendorff et al. 2017, S. 35). Zu Beginn der Corona-Pandemie hat sich die finanzielle Lage gerade der Studierenden stärker angespannt, die aus nicht-Akademiker-Familien stammen, denen ihre Nebenbeschäftigung weggebrochen ist und deren Eltern dies aufgrund eigener finanzieller Einbußen nicht kompensieren konnten (Becker und Lörz 2020).

Die Unterbrechung des öffentlichen Lebens hat Studierende in einer Lebensphase getroffen, die ihre Weiterentwicklung maßgeblich beeinflusst. Der Schritt an die Hochschule, die damit verbundenen (erhofften) Freiheiten und Verantwortungen, die Einbindung in immer wieder neu zusammengesetzte Gruppen, die Veränderung bestehender und das Entstehen neuer Beziehungen stellt sie vor die Chance und Herausforderung, sich äußeren Erwartungen und gesellschaftlichem Rollenverhalten neu anzupassen. Im Laufe des Studiums entsteht so neben dem Erwerb von Wissen und Kompetenzen ein verändertes Bild von sich selbst: Das stete Ausbalancieren zwischen der Außenwelt und der inneren Wirklichkeit trägt zur Identitäts-Entwicklung bei (Bohleber 1999, S. 519). Die Lock-down Maßnahmen, die das studentische soziale Leben abrupt beendet haben, haben diesen Prozess unterbrochen und in völlig andere Bahnen gelenkt. Anstatt herauszugehen, eigene Entscheidungen zu treffen und sich auch im Widerspruch ausprobieren zu können, haben die Studierenden sich rigide anpassen müssen. In der erzwungenen Isolation konnte so das Gefühl entstehen, dass das Leben geschieht, statt aus eigener Initiative gelebt zu werden, ein Zustand, der Phasen der Adoleszenz beschreibt (ebd., S. 521). Gefühle der Hilflosigkeit bewirken eine starke Verunsicherung. Diese wurde durch die Umstellung der Lehre auf online-Vorlesungen, das Angebot von Ersatzleistungen statt Praktika, die Anpassung der Prüfungsformen und Prüfungsordnungen verstärkt. Sie erfolgten ad hoc und letztlich autoritär von den Hochschulen und waren für die Studierenden weder planbar noch vorhersehbar. Einige Studierende blieben alleine am Studienort, andere sind zurück zur Ursprungsfamilie gezogen.

Inwieweit aus den emotionalen Reaktionen auf diese Situation eine anhaltende Beeinträchtigung der seelischen Gesundheit resultiert, hat auch mit der Ausgangslage zu tun. Alle Menschen haben die Stressoren der Pandemie gefühlt, aber die Coping-Strategien haben sich im Zeitverlauf ausdifferenziert. Menschen mit psychischen Vorbelastungen waren in der Covid-Krise besonders verletzlich, der Zugang zu psychiatrischen und psychotherapeutischen Unterstützungsangeboten erschwert (Schedlich 2021, S. 81). Als weitere vulnerable Gruppen wurden u. a. Frauen und junge Menschen ausgemacht (Eichenberg 2021, S. 103). 2016 betrug der Anteil der Studierenden mit psychischen Belastungen deutschlandweit ca. 6 % (Middendorff et al. 2017, S. 36 f.); der Anteil der Studierenden, die darüber berichtet haben, ist in den Ingenieurswissenschaften sowie Rechts- und Wirtschaftswissenschaften geringer als in den Sozial‑, Sprach- und Kulturwissenschaften (ebd.).

Als Schutzfaktoren während der Pandemie und der Lock-down-Phasen gelten stabile Beziehungen, soziale Unterstützung und persönliche Dispositionen sowie eine ausgeprägte Toleranz für Unsicherheit. Dies wurde auch empirisch bestätigt (Eichenberg 2021, S. 104).

## Zur Stichprobe

70 % der Teilnehmer studieren schon länger als 3 Semester, haben also während der Corona-Krise ihr Studium aufgenommen oder sie in Gänze an der Hochschule erlebt; die Alterskohorte 22–25-Jähriger ist am stärksten vertreten (44 %). Von den Befragten haben sich 53 % als männlich, 46,4 % als weiblich und 0,6 % als divers eingeordnet. Da der Anteil männlicher Studierender in allen Fachbereichen außer Wirtschaftsrecht mit bis zu 75 % zum Teil deutlich höher liegt (Westfälische Hochschule 2022) bedeutet dies, dass anteilsmäßig mehr weibliche Studierende an der Umfrage teilgenommen haben.

Insgesamt ist unter den Befragten ein recht hoher Anteil an Langzeitstudierenden (31 % studieren im achten Semester oder länger), älteren Studierenden (knapp 11 % sind 30 Jahre alt oder älter) und Studierenden mit (familiärer) Migrationsgeschichte und/oder aus eher bildungsfernem Elternhaus. 37,5 % der Befragten würde der oder die erste Akademiker/in in der Familie sein. Dies entspricht der Sozialstruktur aller Studierenden an der Westfälischen Hochschule. Auch der hohe Anteil von Studierenden mit Nebenbeschäftigungen (76,7 %) bildet die Lebenswirklichkeit an der Hochschule ab.

## Auswirkungen der Corona-Pandemie und Stimmungsbild heute

Zum Verständnis der Auswirkungen der Corona-Pandemie wurden in Abwandlung der Operationalisierung durch Bering et al. (2021, S. 53 f.) folgende Bereiche abgefragt: 1) die empfundene Bedrohung der Gesundheit, 2) die Gefühlslage während der Pandemie im Vergleich zur Zeit davor, 3) das Studienverhalten und die Studienleistungen, 4) Unterstützung durch nahestehende Personen, 5) Sorgenbelastung und empfundene Nachwirkungen der Corona-Zeit, 6) individuelle Bewältigungsstrategie und 7) eine Frage integriert, welche Unterstützungsangebote gewünscht werden.

### Empfundene Bedrohung der Gesundheit

Von den Befragten sind knapp über 30 % an Corona mit echtem Krankheitsgefühl länger als vier Tage erkrankt, davon 0,85 % mit Krankenhausaufenthalt; die subjektive Bedrohung durch Corona („während der Pandemie hatte ich Angst, an Corona zu erkranken“) lag mit 47,7 auf einer Skala von 0 (gar nicht) bis 100 knapp unter dem Mittelwert. Demgegenüber hat sich auf die Frage „während der Pandemie hatte ich Angst, eine mir nahestehende Person anzustecken“ auf einer Skala von 0 (gar nicht) bis 100 ein Wert von 70 ergeben. Dies korrespondiert mit den Angaben von 59 %, dass ihnen nahestehende Personen an Corona mit echtem Krankheitsgefühl oder Krankenhausaufenthalt erkrankt sind, und 5,9 % der Befragten, die einen durch Corona (mit-) bedingten Todesfall einer nahestehenden Person verkraften mussten.

### Gefühlslage während der Pandemie

Gestresst, einsam, gelangweilt, überfordert, hoffnungslos, ängstlich fühlten sich über 50 % „intensiver/häufiger“ oder „deutlich intensiver/häufiger“. Knapp 49 % erlebten sich als „wütender oder deutlich wütender“. Einsamkeit (71 %) und Stress (63 %) dominierten. Auszüge aus den Freitexten:

„Das Leben wurde schnell eintönig. Man wachte auf, und es war wieder kein Traum, dass dies nun das neue Leben ist, selbst wenn es erstmal ‚nur‘ für eine gewisse Zeit ist. An sich waren die Tätigkeiten nicht das Problem. Einerseits war die Eintönigkeit aber anstrengend und andererseits war die fehlende soziale Interaktion mit unterschiedlichen Personen problematisch. Es tat mit der Zeit weh, ständig nur vor dem Bildschirm zu sitzen und wie jeden anderen Tag auch seine Mitschüler als ausgeschaltete Kameras oder als flache Bilder auf dem Bildschirm zu sehen. Es fiel auf, dass man Menschen braucht, die sich immer wieder ändern. Es soll nicht nur ständig die eigene Familie sein, die man sieht. Es müssen auch mal ganz andere Leute sein. Heute mal zwei Freunde, morgen der Lehrer, übermorgen die Katze, dann die Supermarktverkäuferin. Und jedes Mal ohne den Hintergedanken, dass man gerade in einer halben Dystopie lebt, die von Problemen bestückt ist.“

„Am meisten hat mich gestört, dass ich durch Corona keine Uni-Beziehung aufbauen konnte und es jetzt sehr schwer für mich ist, da die Grüppchen-Bildung eigentlich schon vorbei ist“.

In den Freitexten ergibt sich auch ein konkreteres Bild, wie sich diese Belastungen auf die Seele niedergeschlagen haben. Häufig werden depressive Episoden genannt, teilweise sehr schwer wiegende. Diese werden vorrangig mit der sozialen Isolation und mangelnder Struktur und Überforderung in Verbindung gebracht und wurden dadurch verschärft, dass es sehr schwierig gewesen sei, einen Therapieplatz zu erhalten. Viele Teilnehmer berichten zudem, dass sie Probleme im sozialen Umgang entwickelt hätten. Exemplarisch dazu zwei Stimmen:

„… Dennoch erlebe ich bis heute eine stetige soziale Kälte, die ich vor der Pandemie so nicht kannte und die auch ein Teil von mir selbst geworden zu sein scheint.“

„Ich finde, man hat vor allem jetzt zum Ende der Coronapandemie durch den Krieg in der Ukraine, die Energiekriese und die steigende Inflation ein stärker werdendes Gefühl der Hoffnungslosigkeit, auch dadurch, dass die Coronakrise die Gesellschaft sehr ‚entzweit‘ hat und die so wichtigen zwischenmenschlichen Verbindungen nicht mehr die Qualität besitzen wie vorher.“

Einige Befragte berichten aber auch über Leistungssteigerungen durch das ruhigere Studium von zuhause aus, ein engeres Zusammenrücken innerhalb der Freundesgruppe und Familie und eine allgemein entspannte Situation. Einige wenige Teilnehmer haben keine Änderungen bei sich wahrgenommen.

### Studienverhalten und Studienleistung

Die virtuellen Vorlesungen wurden von 78 % oft oder immer genutzt und von 56 % oft oder immer als hilfreich empfunden. Teilweise hatten sie auch das Potenzial zur Ermutigung, weil sich die Befragten durch die Veranstaltungen oft oder immer aufgebaut gefühlt haben (knapp18 %). Allerdings haben 30 % der Befragten die online-Vorlesung oft oder immer als deprimierend erlebt. Die Studienleistungen haben sich teilweise verbessert (20 %), 43 % haben „teils-teils“ angekreuzt. Einige haben auch gar nichts mehr gelernt (15 %), das Studium unterbrochen (6 %) oder gar innerlich abgebrochen (15 %).

### Unterstützung durch nahestehende Personen

Die meisten Befragten haben Unterstützung durch nahestehende Personen erhalten (85 %) oder selber nahestehenden Personen unterstützt (90 %). In den Freitexten berichten die Teilnehmer von einer Zermürbung im Zeitverlauf, der zunehmenden Anstrengung, die gegenseitige Unterstützung aufrechtzuerhalten. Bei längeren Trennungen habe der regelmäßige virtuelle Austausch geholfen. Insgesamt war die soziale Einbindung hoch.

### Sorgenbelastung und empfundene Nachwirkungen der Corona-Zeit

Während der Corona-Pandemie waren finanzielle Sorgen mit einem Mittelwert von 39 auf einer Skala von null (gar nicht) bis 100 deutlich weniger ausgeprägt als zum Zeitpunkt der Befragung mit einem Mittelwert von 50,8. Auch die allgemeinen Zukunftssorgen haben mit einem Mittelwert von knapp über 60 heute gegenüber der Zeit der Pandemie (58,6) noch einmal zugenommen, wenn auch nicht so deutlich. Gleichzeitig fühlen sich 54 % der Befragten durch die Corona-Krise gegenüber anderen Generationen benachteiligt oder extrem benachteiligt, und 61 % haben das Gefühl, „Dinge verpasst zu haben, die ich nicht mehr nachholen kann“. 26 % sind aus dem Tritt geraten und haben das Gefühl, es nicht mehr aufholen zu können.

Unter den Sorgen dominiert der Studienstress (70 %), gefolgt von allgemeinen Zukunftssorgen und ständigem Grübeln (60 %). Demgegenüber sind die finanziellen Sorgen zwar auch hoch, aber weniger ausgeprägt (40 %), die gesundheitlichen Sorgen (32 %) besorgniserregend. Die Sorgenbelastung ist insgesamt sehr hoch und schlägt sich häufig in Schlafstörungen nieder (41 %). Große oder extreme Sorgen bereiten den Studierenden derzeit die Rahmenbedingungen: Inflation (70 %), Unklarheit insgesamt (66 %), Energiekrise (57 %), Aggressionen in den sozialen Medien (47 %), Ukraine-Krieg (45 %), zunehmende Diskriminierung von Minderheiten (41 %), die Arbeitsmarktsituation (30 %) und die Furcht, dass der Krieg sich zu uns ausbreitet (27 %). Neben weiteren punktuell genannten Themen (u. a. die Kulturrevolution im Iran) beschäftigen vor allem die Klimakrise und das veränderte soziale Klima die Studierenden. Beispielhaft:

„Die bemerkbare soziale Verschlossenheit und die Oberflächlichkeit im Umgang miteinander. Das Gefühl der Machtlosigkeit gegenüber den politischen und klimatechnischen Entwicklungen.“

Teilweise sind die Sorgen sehr breit gefächert:

„Hungersnöte, finanzielle Sorgen, Krieg, Naturkatastrophen, das fehlende Recht auf Meinungsfreiheit, Nationalsozialismus, steigende Kriminalität, Wegwerfgesellschaft, extreme Konsumgesellschaft, extremer täglicher Fleischkonsum/Tierkonsum und deren Folgen auf den Planeten, Atomkriegsgefahr, Autokratie, Diktatur, Hass gegen Menschen, Autobatterie, Gasabkommen mit Nationen aus den arabischen Ländern, Arbeitsplätze.“

Teilweise ist die Befürchtungsdynamik intensiv und diffus:

„Die Angst vor weiteren Problemen, die noch kommen werden. Die Angst, dass es keine wirkliche Ruhe mehr geben wird. Die Angst, dass man mit allem konfrontiert wird, da man kein kleines Kind mehr ist, welches vor allem geschützt wird und welches sowieso mit all dem nichts zu tun hat.“

Ein/e Teilnehmer/in schreibt:

„die unaufhaltbare ökologische Krise, Geld schlägt Moral, Weltschmerz, es geht zu Ende.“

### Bewältigungsstrategien und Schutzfaktoren

Am hilfreichsten wurden während der Corona-Pandemie die sozialen Kontakte zu Freunden (für 57 % sehr oder extrem hilfreich), Familie (50 %) und Liebespartner (45 %) erlebt. Ganz oben rangieren auch Musik hören/Filme schauen (54 %) und ganz praktisch der Wegfall der Fahrtzeiten (54 %). Sport zu treiben war für 38 % sehr oder extrem hilfreich, ihre Haustiere für 27 %. Dann erst folgen die virtuellen Vorlesungen (20 %), der Glaube (11 %) und der Kontakt zu Ansprechpartnern im Fachbereich (7 %). Für die Zeit der Corona-Pandemie werden zusätzlich zu den oben Erwähnten u. a. folgende individuelle Bewältigungsmuster genannt:

„Beispielsweise Mathematik wurde als reines Selbststudium durchgeführt. Mir persönlich hat es gefallen, mich in Bücher zu vertiefen und ‚auf eigene Faust‘ die Inhalte zu begreifen.“

„Alkohol und Drogen“; „Videospiele gerade online mit Freunden haben geholfen.“

„Ich habe bis August 2022 eine Ausbildung als Veranstaltungskauffrau gemacht. Während der Hochphasen der Pandemie wurde unsere Location zum Impfzentrum. Dadurch war ich nicht in Kurzarbeit, habe gearbeitet bzw. hatte in gewissen Maßen meinen Alltag, war ‚aktiv‘ und hatte Kontakt zu Menschen. Das hat mir sehr geholfen.“

Insgesamt geben 50 % der Befragten an, dass es ihnen seit dem Wegfall der Beschränkungen und der Verfügbarkeit der Impfstoffe wieder besser oder sehr viel besser gehe, aber bei vielen wirkt die Zeit noch nach (39,6 %). Manche kommen nicht mehr hoch (8,3 %).

In den Freitexten wird deutlich, dass die Studierenden Freude und Selbstbestätigung jetzt wieder überwiegend im sozialen Austausch erleben, sei es in der Freizeit, sei es an der Hochschule oder im Ehrenamt. Dies dominiert bei Weitem. Aber auch Studienerfolge, das Lernen, Programmieren, Lesen und Musizieren helfen. Viele sind froh über die zurückgewonnene Struktur. Einige haben es noch nicht geschafft, wieder anzuschließen. Sie antworten auf die Frage, was ihnen Freude bereitet: „nichts“ oder berichten von ihrer Therapie. Besondere Aufmerksamkeit verdienen dabei diejenigen, denen es (noch?) nicht gut geht, die aber damit allein bleiben. Stellvertretend für diese Gruppe steht dieses Statement:

„Achtsamkeitsübungen, spazieren, Joggen, Musik hören, Kochen, Backen, Lesen, Spielen, Filme/Serien schauen, Basteln. Aber auch nur wenn ich es schaffe, aus meinem Grübeln rauszukommen. Ansonsten verspüre ich schon lange keine Freude mehr.“

Hoffnung machen den Befragten gelingende soziale Kontakte, die Rückkehr zur Normalität, Studienerfolge zu erleben, ihren Zielen näher zu kommen. Auch technischer Fortschritt ist Anlass zur Hoffnung. Stellvertretend für viele steht dieser Kommentar:

„Aktuelle Bauvorhaben, Gasinfrastruktur für Wasserstoff, viele Stellenangebote für Ingenieure, erneuerbare Energie in bspw. Namibia, und die Erfindung des Corona-Impfstoffs“.

Positiv bewertet werden zudem Entwicklungen, die einen Kulturwandel in der Gesellschaft anstoßen. Neben erlebter Hilfsbereitschaft von Fremden, einem sozialen Umfeld, in dem Diversität und Toleranz fortschreiten, wird mehrfach genannt: „verbreitete Akzeptanz, über psychische Gesundheit zu reden“. Teilweise hilft eine optimistische, gleichwohl reflektierte Grundhaltung, die Ungewissheit zu ertragen:

„Ich versuche mir oft zu sagen, dass solche Krisen auch Chancen in sich tragen und an der ein oder anderen Stelle Entwicklungsschritte gemacht werden, die längst überfällig sind. Außerdem versuche ich ein Verständnis für die viele Ungewissheit zu bekommen und für mich dazuzulernen. Man sagt oft, es wird erst schlimmer, bevor es besser wird, und meiner Auffassung nach sind Krisen, wie wir sie jetzt erleben, auch einfach ein Anzeichen für eine große Veränderung, denn es entsteht oft erst viel Chaos, bevor sich etwas neu sortieren kann.“

Die Antworten bieten insgesamt ein sehr breites Spektrum an Bewältigungsstrategien, aber auch hier sticht eine nicht unerhebliche Anzahl an Kommentatoren heraus, denen „nichts“ einfällt, die schreiben, es gäbe keine Entwicklungen, die ihnen Hoffnung machten. Stellvertretend dafür dieser Beitrag:

„Ich mag die Frage nicht. Die ist deprimierend.“

### Was wünschen sich die Studierenden von uns Ansprechpartnern an der Hochschule?

#### Psychologische Unterstützung

Die Studierenden wünschen sich mehr „Gelegenheit, über Depressionen zu reden“, eine Ausweitung psychologischer Angebote, ein allgemeines Verständnis für die Situation der Studierenden und eine Aufklärung über psychische Erkrankungen, die durch den Corona-Kontext zugenommen haben. Eine Teilnehmerin schreibt:

„Solche Umfragen mehr aufzudrängen, Freiwillige, die solche Studien ausfüllen, sind nicht die betroffene Gruppe. Leute, die extrem beeinträchtigt sind, haben leider kein freiwilliges Interesse, solche Umfragen auszufüllen, basierend auf meinem Umfeld.“

#### Kommunikation auf Augenhöhe

Die Studierenden wünschen eine „bessere Kommunikation auf Augenhöhe“ und mehr Menschlichkeit. Sie wünschen sich weniger soziale Distanzierung und mehr Einfühlsamkeit und Zuwendung.

„Was ich mir von Ansprechpartnern wünsche: Oftmals reichen schon ein paar aufbauende Worte an die Studierenden, was die Zukunft und Jobchancen oder Studienzeit angeht. Das habe ich zuletzt erst wieder in Präsenz erlebt. Man wird doch sehr schnell von dem Stoff erschlagen und verliert ein bisschen den Blick für das große Ganze und macht sich zu viele Gedanken über kleine Details des Stoffes. Mir tut es da immer gut, ab und an wieder geerdet zu werden.“

„Verständnis dafür, dass wir die meiste Zeit nicht an der Hochschule waren und uns gegenseitig in den Kursen teilweise fremd sind. Die ständigen Gruppenarbeiten und Abgaben und der Zwang zur aktiven Teilnahme stressen. Auch die Benotung sowie die Zeitvorgaben sind teilweise zu streng. Ruhigere Studierende werden oft benachteiligt.“

#### Struktur und Anleitung

„Individuelle Unterstützung mit einem Plan, wie man sein Studium und Leben soweit in den Griff kriegt, dass es einem persönlich gut geht.

Ich habe absolut keine Idee, was ich derzeitig machen kann, um meinen Abschluss zu machen, und es gibt keine e‑mails mit Anreizen oder so. […] Es muss einen Leitfaden geben, wie jedem Studierenden erfolgreich geholfen werden kann, um seinen Abschluss zu machen und in die Gesellschaft zu integrieren. […]“

#### Partizipation

„Ich hätte mir in der Corona-Zeit viel mehr Einbindung der Studenten in die Maßnahmen-Politik auch unserer Hochschule gewünscht. Ich empfand es als Frechheit, dass wir nicht gefragt wurden, sondern über unsere Köpfe hinweg entschieden wurde. Wie es damals ja auch Mode war im Allgemeinen. Weniger Framing hätte ich mir auch gewünscht. Dass man nicht jedem politischen Trend folgt. Dass man mehr Menschen hört, die deutlich andere Meinungen vertreten.“

„Ich glaube, das Wichtigste für eine Hochschule ist Transparenz. Ein ehrlicher (und respektvoller) Austausch. Fand es gut, dass es Augenblicke in verschiedenen VL gab, wo ein realistischer und kritischer Blick auf Maßnahmen, Prognosen usw. Würdigung fand.“

Viele Kommentare auf die Frage, was die Studierenden sich wünschen, drücken auch Wertschätzung für die Ansprechpartner vor Ort aus, von denen sie sich gut begleitet und unterstützt fühlen. Fast alle wünschen sich, dass die Hochschule mehr Raum für soziale Interaktionen neben der Lehre bietet. Aber auch auf diese abschließende Frage wird im Antwortspektrum noch einmal deutlich, wie schwierig es für manche bleibt:

„Da bin ich leider überfragt. Ich weiß nicht, was ich brauche, um Wünsche äußern zu können.“

## Diskussion

Mit der Befragung wurde versucht, die in der Literatur diskutierten wesentlichen Entstehungsquellen der (pandemischen) Stressreaktion anzusprechen und auf die Situation von Studierenden zu übertragen. Während der Pandemie haben insbesondere die Isolation und die Befürchtung, nahestehende Personen anzustecken, Stress verursacht. Die Gefühlslage der Mehrheit der Studierenden war entsprechend fragil, was sich auch in ihren Leistungen niedergeschlagen hat. Es hat sich bestätigt, dass viele Studierende in dieser Zeit abgehängt wurden: 21 % der Befragten haben das Studium unterbrochen oder innerlich abgebrochen.

Weiter wird deutlich, dass sich die Sorgenbelastung der Studierenden nach der Pandemie noch erhöht hat, obwohl der externe Druck in Form der erzwungenen Isolation, der Furcht, andere anzustecken, und der erzwungenen Selbstorganisation im Studium weggefallen ist oder abgenommen hat bzw. durch Präsenzangebote seitens der Fachbereiche abgefedert wird.

Insgesamt erscheint die Befürchtungsdynamik hoch. Die während der Pandemie gelernte Hilflosigkeit und Verunsicherung halten an, wirken nach und erschweren das Coping mit der allgemeinen Lebensunsicherheit in einer zunehmend dynamischen und ambiguen Welt. Auch das Gefühl, ohne Schuld abhängt worden zu sein, ist aus vielen Freitexten herauszulesen. Die damit verbundene Resignation fördert Passivität; die Herausforderung, sich äußeren Erwartungen und gesellschaftlichem Rollenverhalten neu anzupassen (s. oben), wird von vielen nicht mehr als belebender Aufbruch zu neuen Ufern empfunden; das veränderte Bild von sich selbst ist teilweise durch die Außenwelt kontaminiert: „Dennoch erlebe ich bis heute eine stetige soziale Kälte, die ich vor der Pandemie so nicht kannte und die auch ein Teil von mir selbst geworden zu sein scheint“ (s. oben).

Es besteht offensichtlich Beratungsbedarf, um sich im Hochschulalltag in bewegten Zeiten zurechtzufinden, u. a. zur Stärkung der Selbstwirksamkeit und Unsicherheitstoleranz. In Anlehnung an Antonovsky gilt es allgemeiner, die Fähigkeit der Studierenden zu stärken, Spannung zu lösen und ihre Transformation in Stress zu verhindern (Antonovsky 1991, S. 118).

Die psychologischen Beratungsstellen der Hochschule reichen nicht mehr aus. Mittelfristig muss es darum gehen, im Sinne eines Hochschul-Gesundheitsmanagements mehrere aufeinander abgestimmte Bausteine zur Unterstützung der Aufrechterhaltung der studentischen Gesundheit anzubieten. Ein Baustein könnte das Angebot eines Gesundheitscoachings für alle Studierende sein, mithin ein Beratungsangebot, das sie darin unterstützt, ihre Studien- und Lebensumstände zu reflektieren und für sie passende Bewältigungsstrategien zu entwickeln (Lewkowicz 2017). Daneben könnten die Studierenden, die ihr Studium unterbrochen oder innerlich abgebrochen haben, gezielt mit Rückkehr-Angeboten angesprochen werden. Diese Empfehlungen auf organisatorischer Ebene sind wohl unabhängig von der in diesem Praxisbericht referierten, sehr eingeschränkten, Datenlage übertragbar auf Hochschulen insgesamt (dazu weitergehend Burian et al. 2023). Ansatzpunkte, wie die Westfälische Hochschule und andere Hochschulen die Studienbedingungen verbessern könnten, lassen sich recht einfach aus den Abfragen herauslesen: mehr soziale Angebote, mehr Struktur und Transparenz sowie Partizipation.

## Footnotes

1 Maschinenbau, Umwelt- und Gebäudetechnik, Elektrotechnik und angewandte Naturwissenschaften, Informatik und Kommunikation, Wirtschaft, Wirtschaft und Informationstechnik, Maschinenbau, Wirtschaftsrecht, Ingenieur- und Naturwissenschaften.
